# Synthesis, DFT Analyses, Antiproliferative Activity, and Molecular Docking Studies of Curcumin Analogues

**DOI:** 10.3390/plants11212835

**Published:** 2022-10-25

**Authors:** Mohamed Jawed Ahsan, Kavita Choudhary, Amena Ali, Abuzer Ali, Faizul Azam, Atiah H. Almalki, Eman Y. Santali, Md. Afroz Bakht, Abu Tahir, Salahuddin

**Affiliations:** 1 Department of Pharmaceutical Chemistry, Maharishi Arvind College of Pharmacy, Jaipur 302 039, Rajasthan, India; 2 Department of Pharmaceutical Chemistry, College of Pharmacy, Taif University, P.O. Box 11099, Taif 21944, Saudi Arabia; 3 Department of Pharmacognosy, College of Pharmacy, Taif University, P.O. Box 11099, Taif 21944, Saudi Arabia; 4 Department of Pharmaceutical Chemistry and Pharmacognosy, Unaizah College of Pharmacy, Qassim University, Uniazah 51911, Saudi Arabia; 5 Addiction and Neuroscience Research Unit, Health Science Campus, Taif University, P.O. Box 11099, Taif 21944, Saudi Arabia; 6 Department of Chemistry, College of Science and Humanity Studies, Prince Sattam Bin Abdulaziz University, P.O. Box 83, Al-Kharj 11942, Saudi Arabia; 7 Department of Pharmacology, Hakikullah Choudhary College of Pharmacy, Ghari Ghat 271 312, Uttar Pradesh, India; 8 Department of Pharmaceutical Chemistry, Noida Institute of Technology (Pharmacy Institute), Knowledge Park-2, Greater Noida 201 306, Uttar Pradesh, India

**Keywords:** antiproliferative activity, curcumin analogues, pyrazole, anti-EGFR

## Abstract

With 19.3 million new cases and almost 10 million deaths in 2020, cancer has become a leading cause of death today. Curcumin and its analogues were found to have promising anticancer activity. Inspired by curcumin’s promising anticancer activity, we prepared three semi-synthetic analogues by chemically modifying the diketone function of curcumin to its pyrazole counterpart. The curcumin analogues (**3a–c**) were synthesized by two different methods, followed by their DFT analyses to study the HOMO/LUMO configuration to access the stability of compounds (∆E = 3.55 to 3.35 eV). The curcumin analogues (**3a–c**) were tested for antiproliferative activity against a total of five dozen cancer cell lines in a single (10 µM) and five dose (0.001 to 100 µM) assays. 3,5-Bis(4-hydroxy-3-methoxystyryl)-1*H*-pyrazole-1-yl-(phenoxy)ethanone (**3b**) and 3,5-bis(4-hydroxy-3-methoxystyryl)-1H-pyrazole-1-yl-(2,4-dichlorophenoxy)ethanone (**3c**) demonstrated the most promising antiproliferative activity against the cancer cell lines with growth inhibitions of 92.41% and 87.28%, respectively, in a high single dose of 10 µM and exhibited good antiproliferative activity (%GIs > 68%) against 54 out of 56 cancer cell lines and 54 out of 60 cell lines, respectively. The compound **3b** and **3c** demonstrated the most potent antiproliferative activity in a 5-dose assay with GI_50_ values ranging between 0.281 and 5.59 µM and 0.39 and 0.196 and 3.07 µM, respectively. The compound **3b** demonstrated moderate selectivity against a leukemia panel with a selectivity ratio of 4.59. The HOMO-LUMO energy-gap (∆E) of the compounds in the order of **3a** > **3b** > **3c**, was found to be in harmony with the anticancer activity in the order of **3c** ≥ **3b** > **3a**. Following that, all of the curcumin analogues were molecular docked against EGFR, one of the most appealing targets for antiproliferative activity. In a molecular docking simulation, the ligand **3b** exhibited three different types of interactions: H-bond, π-π-stacking and π-cationic. The ligand **3b** displayed three H-bonds with the residues Met793 (with methoxy group), Lys875 (with phenolic group) and Asp855 (with methoxy group). The π-π-stacking interaction was observed between the phenyl (of phenoxy) and the residue Phe997, while π-cationic interaction was displayed between the phenyl (of curcumin) and the residue Arg841. Similarly, the ligand **3c** displayed five H-bonds with the residue Met793 (with methoxy and phenolic groups), Lys845 (methoxy group), Cys797 (phenoxy oxygen), and Asp855 (phenolic group), as well as a halogen bond with residue Cys797 (chloro group). Furthermore, all the compound **3a–c** demonstrated significant binding affinity (−6.003 to −7.957 kcal/mol) against the active site of EGFR. The curcumin analogues described in the current work might offer beneficial therapeutic intervention for the treatment and prevention of cancer. Future anticancer drug discovery programs can be expedited by further modifying these analogues to create new compounds with powerful anticancer potentials.

## 1. Introduction

**Figure 1:**
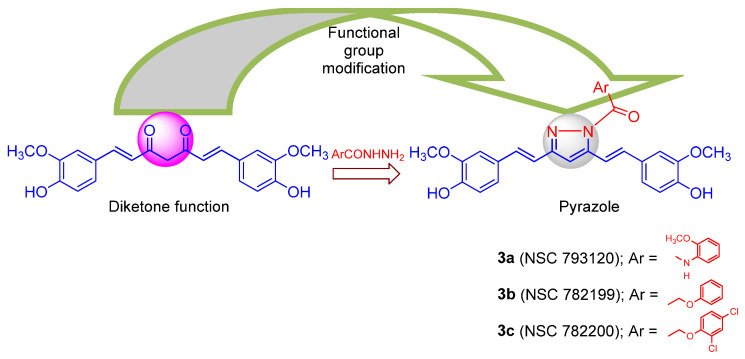
Structural modification of diketone function into pyrazole.

The development of new antiproliferative agents with greater efficacy and a smaller amount of side effects remains a modern scientific research challenge. Over the last few decades, there has been a surge in interest in using natural products for therapeutic purposes. Although this concept appears to be new, our forefathers have used natural compounds/extracts to treat illness since time immemorial. The development of synthetic pharmaceuticals over the last century not only revolutionized modern medicine, but also accrued the undesirable properties and side effects associated with these drugs. The un-favorable properties of synthetic drugs have prompted a search for natural alternatives, with the hope that naturally occurring compounds will be better tolerated and safer than their synthesized counterparts [1,2]. Curcumin, one of the curcuminoids obtained from the powdered root of turmeric (*Curcuma longa Linn.*), belongs to the family *Zingiberaceae* is a β-diketone that showed an antiproliferative effect on various panels of cancer cell lines [3]. Medicinal chemists have identified four main sites to bring about chemical modification in curcumin to form semi-synthetic congeners. The four main sites including the active methylene (-CH_2_-), aryl side chain, diketone group, and carbon-carbon double bonds (-CH=CH-) to create a number of semi-synthetic curcumin analogues with improved bioactivity [4]. In this study, we report on the modification of the diketone group to pyrazole heterocycle and their antiproliferative activity. The chemical modification is outlined in Figure 1. The structural modification was discovered to improve biological activities by increasing stability, decreasing rotational freedom, and minimizing metal-chelation properties [5]. Curcumin analogues have previously been shown to have anticancer, antimalarial, and anti-HIV activities, according to our research team [6,7,8,9,10]. However, a wide range of biological activities have been reported, including antibacterial, anticancer, antioxidant, antimalarial, anti-inflammatory, anti-Alzheimer’s, and anti-HIV activities [11,12,13,14,15,16,17]. Curcumin analogues have also been identified as epidermal growth factor receptor (EGFR) inhibitors [18,19].

Cancer is a disease in which a few of the body’s cells grow out of control and spread to other parts of the body. With 19.3 million new cases and almost 10 million deaths in 2020, cancer has become a leading cause of death today. Today, breast cancer is the most commonly diagnosed cancer, followed by lung cancer. Breast, lung, colorectal, liver, and stomach cancers account for 11.7, 11.4, 10, 7.3, and 5.6 percent of all new cancer cases, respectively [20]. There are over 100 different forms of cancer. According to World Health Organization (WHO) reports, lung cancer is the main cause of cancer-related death in men, while breast cancer is the leading cause of cancer-related death in women. Chronic infections, mostly caused by the human papillomavirus (HPV) and hepatitis B virus (HBV), cause around 20% of all malignancies worldwide, and are avoidable with very efficient vaccines [21]. Tobacco use alone is responsible for 25% of cancer deaths worldwide [22]. Eliminating or reducing exposure to risk factors can prevent one-third to one-half of all malignancies [21]. The treatments of cancer nowadays involve surgical removal and radiation of large accumulated masses followed by systemic chemotherapy [23]. Chemotherapy continues to be a fundamental regime in clinical handling of all types of cancer, although it contains a high risk of toxicity and multidrug resistance (MDR) against anticancer agents [24,25]. As a result, our reliance on nature became more rational, as active constituents of natural origin would be assumed to be safe. Natural products (NPs) derived from plants, marine organisms, and microorganisms account for the vast majority (more than 60%) of anticancer drugs currently in clinical use [23,26]. Plant-derived anticancer drugs such as camptothecin, etoposide, paclitaxel, vinblastine, and vincristine are a few examples that are currently being used to treat a variety of cancers [27]. Many prospective phytoconstituents have received a lot of attention in the literature for treating conditions including cancer (breast and skin), oxidative stress, inflammation, etc. [28,29]. Curcumin, a β-diketone derived from the powdered root of turmeric, has been shown to have an antiproliferative effect on a variety of cancer cell lines; however, bioavailability is a major issue [3,30,31]. Chemical modification of functional groups could be used to alter curcumin bioavailability, and we converted the diketone function into its pyrazole counterpart in the current study to prepare their semi-synthetic analogues. Several studies have shown that this type of modification increases biological activity [5].

The EGFR is a key anticancer target and the most studied receptor in the tyrosine kinase super-family [32,33]. Curcumin and their semi-synthetic congeners were found to have anti-EGFR activity [7,18]. We previously reported that the bulky nature of curcumin analogues allows them to fit well within the active site of EGFR and exhibit a variety of interactions such as H-bonding, π-π-stacking, and π-cationic. They demonstrated interaction with important EGFR active site residues such as Cys797, Met793, and Arg841 [7]. We investigated the antiproliferative and anti-EGFR activities of newer curcumin analogues in this study.

## 2. Results

### 2.1. Chemistry

**Scheme 1:**
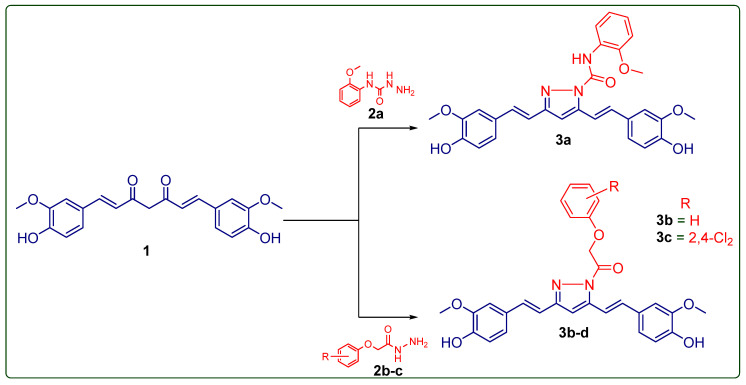
Protocol for the synthesis of semi-synthetic curcumin analogues (**3a–c**).

The curcumin analogues (**3a–c**) synthesized in the present investigation are summarized in Scheme 1 and two different methods (Method A and Method B) were adopted for the synthesis. 3,5-Bis-4-hydroxy-3-methoxystyryl)-N-(2-methoxyphenyl)-1H-pyrazole-1-carboxamide (**3a**) was prepared by heating an equimolar mixture of curcumin (**1**) (0.001 mol; 368 mg) and N-(2-methoxyphenyl)hydrazine carboxamide (**2a**) (0.001 mol; 181 mg) in glacial acetic acid at 80 °C. Similarly, 1-(3,5-bis((E)-4-hydroxy-3-methoxystyryl)-1H-pyrazol-1-yl)-2-(substituted phenoxy)ethan-1-ones (**3b–c**) were prepared by heating an equimolar mixture of curcumin (1) (0.001 mol; 368 mg) and substituted phenoxy-acetohydrazide (**2b–c**) in glacial acetic acid at 80 °C. In this method (Method A), the reaction mixture was heated with continuous stirring for 10 h to complete the reaction while thin layer chromatography (TLC) in n-hexane: ethylacetate (6:4) was used to monitor the reaction throughout. The curcumin analogues were also prepared by another method (Method B). The method B glycerol-water system (1:2) was taken as solvent and the reaction was charged at 60–80 °C with continuous stirring for 6 h. The yields and time taken in the reaction are compared in Table 1. The method B was found to be more promising for the synthesis of curcumin analogues (**3a–c**). The synthetic protocol is summarized in Scheme 1. The intermediates **2a**, and **2b–c** were prepared using the methods described elsewhere [34,35]. The structure of curcumin analogues (**3a–c**) were confirmed by spectroscopic techniques. The IR spectra of curcumin analogues **3b–c** showed acyl carbonyl (-CH_2_C=O) peak at 1650 and 1600 cm^−1^, while pyrazole C=N of compounds **3a–c** was observed at 1544 to 1548 cm^−1^. The phenolic function (ArOH) of compounds **3a–c**, while the phenoxy (-O-) functions of compounds **3b–c** were observed ranging between 3427–3431 and 1276–1280 cm^−1^, respectively. The peak of the C-Cl function of **3c** was observed at 680 cm^−1^. The ^1^H NMR showed a singlet for the six protons the methoxy function (OCH_3_) of curcumin in compounds **3a–c** at δ 3.82–3.83 ppm, while the methoxy function (OCH_3_) present in the phenyl ring of compound **3a** at δ 3.69 ppm. The methylene bridge (-CH_2_-) function of compounds **3b** and **3c** were observed at δ 4.60 and 4.80 ppm, respectively. The pyrazole CH of compounds **3a–c** was observed as a singlet at 6.61 ppm and a broad singlet for the two protons of phenolic function (ArOH) at δ 9.14–9.91 ppm. The secondary amine peak of compounds 3a was observed as a singlet at δ 10.56 ppm. Two doublets were observed at δ 6.73–6.77 ppm corresponding to the -CH=CH- with coupling constant (*J*) of 12.4–14.4 Hz confirming the *trans* couplings. The aromatic protons of compounds **3a–c** were observed as a singlet, and multiplet depending upon the nature of protons at δ 7.80–7.56 ppm. The ^13^C NMR of the prototype compound **3b** displayed different carbon peaks at δ 160.71, 151.32, 147.46, 144.89, 137.31, 133.41, 129.83, 128.81, 123.6, 121.18, 120.19, 116.80, 114.31, 112.101, 107.72, 72.09, and 56.41 ppm. The ES-MS spectra of compound **3b** showed a peak at *m*/*z*, 498.1 and 499.1 corresponding to M^+^ and (M + 1)^+^ corresponding to the molecular formula C_29_H_26_N_2_O_6_.

### 2.2. DFT Studies

Density functional theory (DFT) studies are critical for comprehending intermolecular dynamics and designing molecules with desired pharmaceutical characteristics. This tool may be used to identify a molecule’s fundamental properties such as its frontier molecular orbital energy levels, chemical reactivity, and stability, among others [36,37]. To further understand the impact of the molecular fragments, DFT calculations were conducted using the B3LYP/6-311G(dp) basis set. The DFT-optimized conformations of the compounds **3a–c** have been presented in Figure S1 [Supplementary Materials]. 

As shown in the Figure S2 [Supplementary Materials], HOMO is mostly confined to one of the 4-hydroxy-3-methoxyphenyl fragments attached to the pyrazole functionality, with appreciable contributions from the latter. LUMO, on the other hand, is mostly restricted to the pyrazole ring. However, the aromatic ring appended through the amide bond in the pyrazole ring was not covered by either HOMO or LUMO in the DFT-optimized molecules. The HOMO and LUMO energies and relevant global reactivity descriptors are presented in Table 2. The HOMO-LUMO gap for the compounds **3a–c** was determined to be 3.55, 3.40 and 3.35 eV, respectively. This is a typical value for tiny organic cores and corresponds to the prior study [38].

### 2.3. Antiproliferative Activity

The antiproliferative activity of the curcumin analogues was carried out against five dozen cancer cell lines derived from nine diverse panels including, breast, colon, CNS, leukemia, melanoma, non-small cell lung, ovarian, renal, and prostate cancer cell lines at one dose (10 µM) and five dose assay (0.01, 0.10, 1.00, 10, and 100 µM), as per the National Cancer Institute US [39,40,41,42]. The anticancer activity is represented as growth percent (GP) and percent growth inhibition (%GI), and are related as: %GI = 100 − GP. In single dose assay the compound **3a** displayed less antiproliferative activity and was found to displayed moderate activity against NCI-H522 and HT29 with growth percent (GP) of 73.19 and 80.40 percent, respectively. The compound **3b** displayed maximum anticancer activity with growth percent (GP) 7.59 percent (percent growth inhibition; %GI = 92.41%) followed by compound **3c** (GP = 12.72%; %GI = 87.28%) and **3a** (GP = 99.22%; %GI = 0.78%). The curcumin analogues **3b** and **3c** were found to be highly sensitive against all the leukemia cancer lines. The antiproliferative data of curcumin was retrieved from the NCI data base with NSC 32982 [39]. The compound is supposed to be active on particular cell lines if the GP is found to be 32% or less (i.e., %GI = 68% or more) [40,41,42,43,44]. The compound **3c** showed a lethal effect on leukemia cell lines HL-60 (TB) (%GI = 118.70%) and RPMI-8226 (%GI = 106.80%), while the compound **3b** displayed a lethal effect on RPMI-8226 (%GI = 103.54%). Similarly, the compounds **3b** and **3c** displayed a lethal effect on non-small lung cancer cell lines NCI-H522 with %GIs of 139.28 and 128.71, respectively. The compound **3b** and **3c** displayed more antiproliferative activity than curcumin against non-small lung cancer cell lines panel. The compounds **3b** and **3c** were found to be active against all colon cancer cell lines except COLO205 and HCC-2998 and a lethal effect was observed on the colon cancer cell line HT29 with %GIs of 133.23% by the compound **3b**. The compounds **3b** and **3c** were also found to be active on all the CNS cancer cell lines, while lethal effect was observed against SF-539 (%GI = 110.32%) by compound **3b**. The compounds **3b** and **3c** displayed more promising antiproliferative activity than curcumin against most of the colon and CNS cancer cell lines. The compound **3c** exhibited a lethal effect on melanoma cell lines MDA-MB-435, SK-MEL-2, and SK-MEL-5 with %GIs of 112.15, 104.36 and 112.95 percent, respectively, while the compound **3b** exhibited a lethal effect on melanoma cell line SK-MEL-2, and SK-MEL-5 with %GIs of 118.73, and 128.80, respectively. The compounds **3b** and **3c** were found to be active against all the melanoma cancer cell lines and also displayed superior antiproliferative activity to curcumin. The compound **3b** exhibited a lethal effect on ovarian cancer cell lines OVCAR-3 and SK-OV-3 with %GIs of 104.46 and 128.58%, respectively. The compounds **3b** and **3c** also displayed superior antiproliferative activity to curcumin against ovarian cancer cell lines. The curcumin analogues **3b** and **3c** displayed promising antiproliferative activity against melanoma and ovarian cancer panels and their activity was found to be superior to curcumin. The compound **3b** and **3c** displayed a lethal effect on renal cancer cell lines with %GIs of 144.52 and 109.98 percent, while the compound **3b** showed a lethal effect on breast cancer cell lines BT-549 and MDA-MB-468 with %GIs of 103.68 and 101.76 percent. Both the compounds were found to be active against all the cell lines of renal, prostate and breast cancer cell line panels with %GIs > 68 percent and displayed superior antiproliferative activity to curcumin. The mean GPs and %GIs showed that the curcumin analogues **3b** and **3c** showed superior antiproliferative activity to curcumin as shown in Figure S3 (leukemia, non-small cell lung cancer, colon and CNS cancer panels) and Figure S4 (melanoma, ovarian, renal prostate and breast cancer panels) [Supplementary Materials]. The antiproliferative activity of all the compounds (**3a–c**) in term %GIs is given in the Table 3, while the antiproliferative activity of all the compounds (**3a–c**) in term GP is given in the Table S1 [Supplementary Materials]. Furthermore, the compound 3a demonstrated maximum sensitivity against NCI-H522 with GP of 73.19% (%GI = 24.81%) and least sensitivity against IGROV1 with GP of 109.32% (%GI = −9.32%). The compound 3b demonstrated maximum sensitivity against RXF 393 with GP of −44.52% (%GI = 144.52%) and least sensitivity against COLO205 with GP of 79.25% (%GI = 20.75%). Similarly, the compound **3c** demonstrated maximum sensitivity against NCI-H522 with GP of −28.71% (%GI = 128.71%) and least sensitivity against COLO205 with GP of 82.37% (%GI = 17.63%). The curcumin analogues **3b** and **3c** displayed promising antiproliferative activity in the one dose assay (10 µM). The compound **3b** exhibited good inhibitions against 54 cancer cell lines out of 56 cancer cell lines, similarly the compound **3c** exhibited good inhibitions against 54 cancer cell lines out of 60 cancer cell lines at 10 µM. The antiproliferative data of curcumin analogues (**3a–c**) in a single dose assay at 10 µM are given in Figures S5–S7 (Supplementary Materials). Because the compounds (**3b** and **3c**) demonstrated promising antiproliferative activity in a single dose assay, they qualified for 5-dose assay testing [40,41,42,43,44].

**Figure 2:**
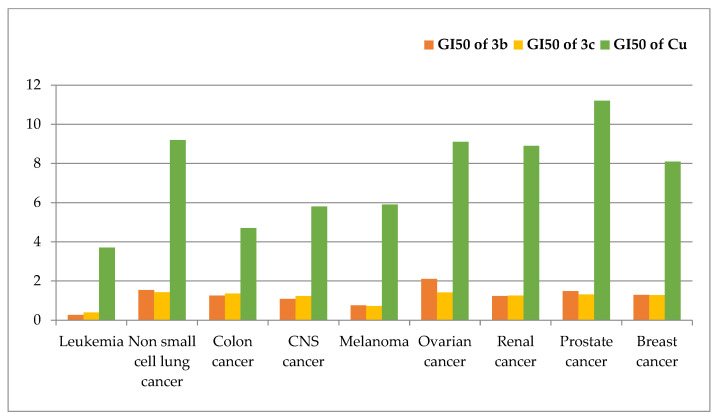
The mean GI_50_ (in µM) for curcumin (Cu) and curcumin analogues **3b** and **3c**.

In the 5-dose assay the compound **3b** demonstrated promising antiproliferative activity against 60 NCI cell lines with GI_50_ values ranging between 0.281 and 5.59 µM, TGI values ranging between 0.49 and >100 µM, and LC_50_ values 4.48 and >100 µM. Similarly, the compound **3c** exhibited promising antiproliferative activity with GI_50_ values ranging between 0.224 and 3.82 µM, TGI values ranging between 0.837 and >100 µM, and LC_50_ values 11.4 and >100 µM. The antiproliferative activity of compounds **3b** and **3c** against 60 NCI cancer cell lines in the 5-dose screening in terms of GI_50_, TGI, and LC_50_ are given in Table 3. The compound **3b** displayed the most promising antiproliferative activity against CCRF-CEM (GI_50_ = 0.281 µM), while the compound **3c** displayed the most promising activity against SR (GI_50_ = 0.224 µM) among the leukemia cell lines. The compound **3b** displayed the most promising antiproliferative activity against HOP-62 (GI_50_ = 1.08 µM), while the compound **3c** displayed the most promising activity against NCI-H460 (GI_50_ = 0.517 µM) among the non-small lung cancer cell line. The compound **3b** displayed the most promising antiproliferative activity against HCT-116 (GI_50_ = 0.386 µM), while the compound **3c** displayed the most promising activity against HCT-15 (GI_50_ = 0.342 µM) among the colon cancer cell line. The compounds **3b** and **3c** displayed the most promising antiproliferative activity against the CNS cancer cell line SF-539 with GI_50_ values of 0.354 and 0.38 µM, respectively. The compounds **3b** and **3c** displayed the most promising antiproliferative activity against the melanoma cancer cell line MDA-MB-435 with GI_50_ values of 0.21 and 0.243 µM, respectively. The compounds **3b** and **3c** displayed the most promising antiproliferative activity against the ovarian cancer cell line OVCAR-3 with GI_50_ values of 0.552 and 0.511 µM, respectively. The compound **3b** and **3c** displayed the most promising antiproliferative activity against the renal cancer cell line UO-31 with GI_50_ values of 0.302 and 0.362 µM, respectively. The compounds **3b** and **3c** displayed the most promising antiproliferative activity against the prostate cancer cell line DU-145 with GI_50_ values of 1.37 and 0.833 µM, respectively. The compounds **3b** and **3c** displayed the most promising antiproliferative activity against the breast cancer cell line MCF7 with GI_50_ values of 0.343 and 0.336 µM, respectively. The compound **3b** exhibited less selectivity against all the eight panels (except leukemia) of cancer cell lines with a selectivity ratio (SR) ranging between 0.59 and 1.63, similarly the compound **3c** exhibited less selectivity against all the panels of cancer cell lines with SR ranging between 0.80 and 2.94 (Table 3). The mean GI_50_ for an individual panel was calculated for each curcumin analogues (**3b** and **3c**) (Table 3; Subpanel MID^a^) and compared with that of the curcumin (Figure 2). The curcumin analogues **3b** and **3c** displayed superior activity to curcumin (Figure 2). The curcumin analogues displayed superior antiproliferative activity to curcumin. Furthermore, the compounds **3b** exhibited moderate selectivity against the leukemia panel of cancer cell lines with a selectivity ratio of 4.59. The value of SR > 6 showed higher selectivity, SR with 3–6 value showed moderate selectivity, while the SR value less than 3 showed less selectivity against a particular panel of cancer cell lines [45,46]. The anticancer data for compounds **3b** and **3c** in terms of TGI and LC50 in µM concentrations is given in Table S1 (Supplementary Materials) and a graph plot between GP and Log10 concentrations are given Figure S8 (for compound 3b) and Figure S9 (for compound 3b) (Supplementary Materials).

**Figure 3:**
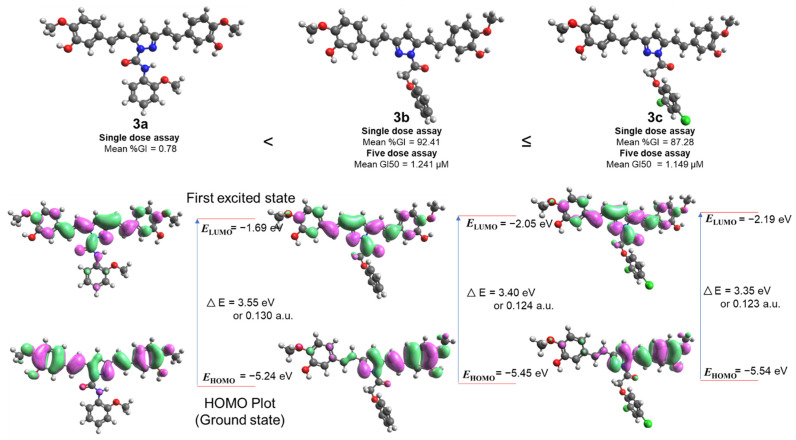
DFT-optimized structures, HOMO/LUMO visualization, anticancer activity and DFT correlation of compounds **3a–c**.

According to the frontier-orbital theory, the two major parameters that influence bioactivities are the highest occupied molecular orbital (HOMO) and the lowest unoccupied molecular orbital (LUMO) [47,48,49]. Thus, the study of the frontier-orbital energies may be helpful to the investigation of anticancer activity. The HUMO-LUMO gaps (∆E) of the three compounds followed as **3a** > **3b** > **3c**. The narrow HUMO-LUMO gap (∆E) implies a high chemical reactivity as well as biological activity [50]. This suggested that compound **3c** might possess a relatively high anticancer activity. The anticancer activity of **3b,c** was found to be promising in a single dose assay with %GIs of 92.41 and 87.28, respectively, at 10 µM, while the mean GI_50_ was calculated as 1.241 and 1.149 µM, respectively, in the five dose assay. Both the compounds were found to be active against 54 cancer cell lines. Comparing the anticancer activity of the three compounds, the order followed as **3c** ≥ **3b** > **3a** (Figure 3).

### 2.4. Molecular Docking Studies

**Figure 4:**
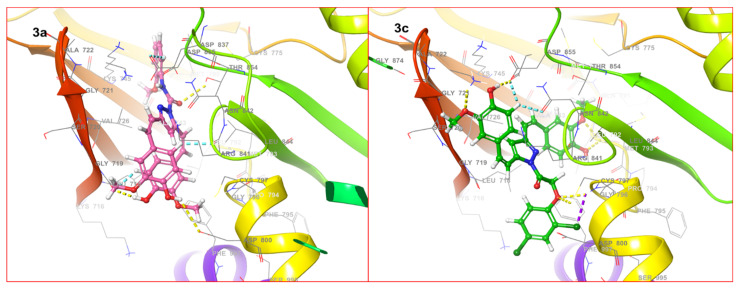
The 3D interactions of ligands **3a,c** against the active site of EGFR.

**Figure 5:**
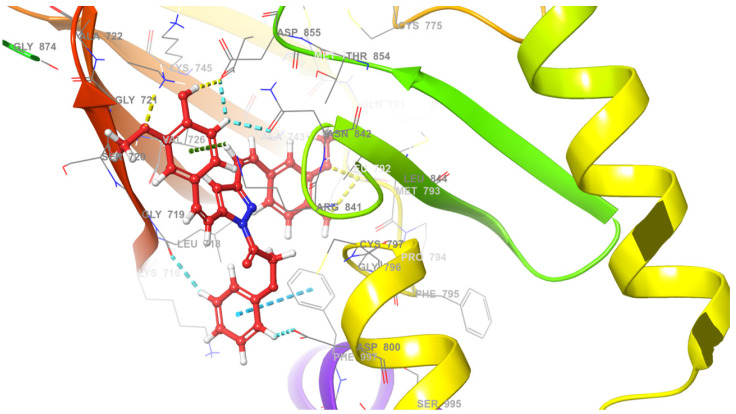
The 3D interactions of ligand **3b** against the active site of EGFR.

The anti-EGFR activity of curcumin and curcumin analogues was well documented in the literature [7,18]. The molecular docking against EGFR (PDB ID: 3W2R) was carried out as a putative mechanism of anticancer activity of the curcumin analogues investigated in the current work as per the reported protocol [51]. The molecular docking score, 2D interaction images, and types of interactions of curcumin analogues and curcumin are given in Table 4. All the curcumin analogues displayed efficient binding against the active site of EGFR with a binding affinity of −6.003 to −7.957 kcal/mol, while curcumin displayed a binding affinity of −7.391 kcal/mol. The ligand **3a** displayed two H-bonds one between the phenolic function and the residue Leu718 and another between the carbonyl function and residue Thr854. The ligand **3c** displayed four H-bonds and a halogen bond, the two H-bonds between one of the phenolic and methoxy functions and the most important residue Met793, while one H-bond was present between one of the phenolic functions and the residue Asp855 and one H-bond between the methoxy function and the residue Lys875. The 3D interaction of the ligands (**3a,c**) is shown in Figure 4. The ligand **3b** was found to be the most active compound in the series exhibiting three different types of interactions, including H-bond, π-π-stacking and π-cationic interactions. The ligand **3b** displayed three H-bonds, one H-bond between the methoxy function and the most important residue Met793, one H-bond between another methoxy function and the residue Lys875 and one H-bond between the phenolic function and the residue Asp855. The π-π-stacking interaction was observed between phenyl (of phenoxy) and the residue Phe997, while π-cationic interaction was displayed between phenyl (of curcumin) and the residue Arg841. The 3D interactions of ligand **3b** against the active site of EGFR are shown in Figure 5. The curcumin displayed two types of interactions: H-Bond interaction with the residues Phe856, Ala743, andLys745 and π-Cationic with the residue Arg858.

## 3. Discussion

The anticancer activity of six 3,5-bis(4-hydroxy-3-methylstyryl)-N-(substitutedphenyl)-1H-pyrazole-1-carboxamides has previously been reported, and their antiproliferative properties in terms of GPs ranged between 7.23 and −19.19 percent, which were found to be higher than that of compound **3a**, which showed a mean percent GP of 99.22 percent [7]. The curcumin analogues reported earlier showed superior antiproliferative activity to curcumin in the one dose (10 µM) as well as the five dose assay [7,52]. The curcumin analogue displayed maximum anticancer activity with IC_50_ of 7.1 µM against MCF7 cell line [53]. The curcumin pyrazole reported by other researchers displayed cytotoxicity against PC-3, MCF-7, MDA-MB-231 with IC_50_ values of 5.6 ± 2.0, 5.9 ± 0.6 and 6.6 ± 1.9 µM, respectively [54]. The curcumin analogues reported displayed anticancer activity against Hep-G2, HCT-116, and QG-56 cell lines with IC_50_ values ranging between 12.5 and 50 µM [55]. The cucumin analogue showed maximum cytotoxicity against LNCaP and PC-3 with IC_50_ values of 54.8 ± 2.5 and 52.1 ± 4.8 µM, respectively [56]. The curcumin analogue showed cytotoxicity against MCF-7, MDA-MB-231, HeLa, DU-145, and SKNSH with IC_50_ values of 5.31, 8.33, 7.69, 8.62, and 8.19 µM and also inhibited Akt and STAT3 phosphorylation and increased ERK phosphorylation [57]. The curcumin analogue inhibited tubulin with an IC_50_ value of 16 µM [58]. The curcumin analogues (**3b** and **3c**) in the current investigation showed superior antiproliferative activity to curcumin in the one dose as well as the five dose assay. The curcumin analogues (**3b,c**) displayed maximum sensitivity against the leukemia cell line panel with GI_50_ values ranging between 0.224 and 0.569 µM (Table 3), while the sensitivity ratios were found to be 4.59 and 2.94, respectively. A series of four curcumin analogues have also been reported by our research team that showed antiproliferative activity with a mean GP ranging between 0.92 and −16.09, while compounds (**3b,c**) displayed mean GPs of 7.59 and 12.72, respectively [52]. The chemical modifications of a diketonic function into pyrazole and dihyroprimidinone analogues were always found to be more promising when compared with the chemical modification of a diketonic function into bigenelli type curcumin analogues [7,10,55,59,60]. The curcumin analogues reported in another published work demonstrated cytotoxicity on the CCGF-CEM cell line with IC_50_ ranging between 3.13 and 93.40 µM, while compounds **3b** (GI_50_ = 0.281 µM) and **3c** (GI_50_ = 1.34 µM) in the current investigation showed superior activity against the same cell lines [61]. The compounds 3,5-Bis(4-hydroxy-3-methoxystyryl)-1H-pyrazole-1-yl-(substituted phenoxy)ethanones (**3b,c**) were reported for the first time in the current investigation and they demonstrated promising antiproliferative activity against 5 dozen cancer cell lines derived from nine diverse panels. Some of the curcumin analogues reported earlier showed IC50 values of 16.71 (curcumin pyrazole) and 5.85 µM (curcumin semicarbazide) in an SRB assay against the HCT 116 cell line, while our compounds **3b** and **3c** displayed superior anticancer activity with GI_50_ values of 0.386 and 0.416 µM, respectively [62]. The curcumin analogue **3b** was found to be moderately selective against the leukemia panel with an SR of 4.59. In our previous work the curcumin analogues were found to be non-selective towards the cancer cell line panels [7]. In the current study, it was discovered that the curcumin analogues demonstrated better antiproliferative activity than previously reported curcumin analogues [63,64]. Since the reported curcumin analogues (**3a–c**) in the current investigation showed encouraging binding affinity against the EGFR, this information may also help to highlight the biological significance of curcumin analogues.

## 4. Materials and Methods

### 4.1. Preparation of Curcumin Analogue ***3a***

An equimolar mixture of curcumin (**1**) (1 mmol; 368 mg) and N-(2-methoxyphenyl)-hydrazine carboxamide (**2a**) (1 mmol; 181 mg) in glacial acetic acid was stirred continuously at 80 °C in a sand bath for 10 h. The reaction mixture was then concentrated under a vacuum to remove excess solvent before being poured into crushed ice, filtered, dried, and recrystallized with ethanol to yield the compound **3a**. The intermediate **2a** was prepared as per the reported method in two steps starting from o-anisidine [34]. Method A used glacial acetic acid as a solvent, whereas Method B used a green solvent system glycerol-water (1:2), which was found to be faster and yielded slightly more.

### 4.2. Preparation of Curcumin Analogue ***3b,c***

An equimolar mixture of curcumin (**1**) (1 mmol; 368 mg) and substituted phenoxy acetohydrazide (**2b–c**) (1 mmol) in glacial acetic acid was stirred continuously at 80 °C in the sand bath for 10 h. The reaction mixture was then concentrated under a vacuum to remove excess solvent before being poured into crushed ice, filtered, dried, and recrystallized with ethanol to yield compound **3b–c**. The intermediates **2b–c** were prepared as per the reported method in two steps starting from substituted phenol as per the reported method [35]. Method A used glacial acetic acid as a solvent, whereas Method B used a green solvent system glycerol-water (1:2), which was found to be faster with slightly higher yields.

### 4.3. DFT Analyses

The ligand’s 2D structure was created in Marvin Sketch (Marvin, 21.14, 2021, ChemAxon, http://www.chemaxon.com/ accessed on 24 July 2022), then transformed to a 3D structure and saved in xyz format. The DFT calculations were carried out in the gas phase using the Orca 5.03 package [65,66] and the B3LYP/6-311G (dp) basis set [67]. Chemcraft (https://www.chemcraftprog.com accessed on 24 July 2022) and Avogadro programs were used for analysis of the DFT results [68,69]. 

### 4.4. Antiproliferative Activity

The antiproliferative activity of the curcumin analogues (**3a–c**) was evaluated against nine diverse panels of 60 cancer cell lines at 10 µM according to the National Cancer Institute (NCI US) protocol [39,40,41,42]. The antiproliferative activity was calculated as growth percent (GP) and percent growth inhibition (%GI) at one dose assay at 10 µM. In the five dose assay, the curcumin analogues (**3b–c**) were treated against the cell lines in the given concentrations of 0.001 to 100 µM and there different parameters viz. GI_50_, TGI and LC_50_ were calculated for each cell line [43,44]. The parameters GI_50_, TGI and LC_50_ are the molar concentration producing 50% growth inhibition, total growth inhibition (TGI) and a 50% cellular death, respectively.

### 4.5. Molecular Docking Studies

The ligands 3a-c were molecular docked against EGFR. The protein data bank provided the EGFR (PDB: 3W2R) X-ray crystal structure with a resolution of 2.05 Å; R-value 0.220 (observed) (https://www.rcsb.org/structure/3W2R accessed on 24 July 2022) [70]. The ligands **3a–c** were saved as mol files, and docking was performed according to the reported protocol [7,51].

## 5. Conclusions

We have prepared and reported the antiproliferative activity of three curcumin analogues (**3a–c**). The antiproliferative activity of compounds **3b** and **3c** showed promising anticancer activity in a one dose as well as a five dose assay. The compound **3b** showed the most promising antiproliferative activity among the series of curcumin analogues and showed moderate selectivity against leukemia with an SR value of 4.59. The curcumin analogues (**3b,c**) demonstrated superior antiproliferative activity to curcumin. HOMO-LUMO gaps (∆E) from DFT analysis and anticancer activities were discovered to be in agreement. All the curcumin analogues (**3a–c**) demonstrated significant binding affinity against the EGFR, a potential anticancer target. Because the compounds demonstrated promising anticancer activity and significant binding affinity against the EGFR, the current report has the potential to enhance the anticancer research development program in the future.

## Figures and Tables

**Table 1:** The percentage yield and reaction time for the synthesis of curcumin analogues (**3a–c**).

S. No.	Compound	Method	Reaction Condition	Yield (%)	Reaction Time (in h)
1	**3a**	Method A	Stirring at 80 °C in AcOH	72	10
2	**3b**			78	10
3	**3c**			74	10
4	**3a**	Method B	Stirring at 60–80 °C in Glycerol-water system (1:2)	75	6
5	**3b**			80	6
6	**3c**			78	6

**Table 2:** DFT results for compounds **3a–c** in terms of global reactivity descriptors.

Parameters	3a	3b	3c
HOMO	−5.242	−5.445	−5.540
LUMO	−1.693	−2.049	−2.193
HOMO-LUMO gap	3.549	3.396	3.347
Hardness (*η*)	1.775	1.698	1.674
Softness (*S*)	0.564	0.589	0.598
Chemical potential (*μ*)	−3.468	−3.747	−3.867
Electronegativity (*χ*)	3.468	3.747	3.867
Electrophilicity index (*ω*)	3.388	4.134	4.467

**Table 3:** 60 NCI cancer cell lines-based antiproliferative activity of curcumin analogues **3a–c** in single dose (10 µM) and 5-dose assay (0.001–100 µM) of curcumin analogues **3b–c**.

Panel	Cell Line	Single Dose Assay (10 µM)			Five Dose Assay					
		3a	3b	3c	3b			3c		
		%GI	%GI	%GI	GI_50_	MID ^b^	SR	GI_50_	MID ^b^	SR
***Leukemia***	CCRF-CEM	4.17	**93.21**	**95.65**	0.281	0.27	4.59	0.432	0.39	2.94
	HL-60(TB)	14.61	**-**	**118.70**	0.282			0.314		
	K-562	−6.41	**-**	**95.59**	0.366			0.381		
	MOLT-4	7.64	**-**	**100.33**	0.368			0.425		
	RPMI-8226	−2.92	**103.54**	**106.80**	0.377			0.569		
	SR	1.88	**-**	**99.99**	0.318			0.224		
***Non-Small Cell Lung Cancer***	A549/ATCC	6.89	**91.32**	**89.42**	2.42	1.54	0.81	1.31	1.43	0.80
	EKVX	−0.71	**71.88**	61.65	2.53			1.98		
	HOP-62	8.37	**91.61**	**88.72**	1.08			0.519		
	HOP-92	−4.08	**85.63**	**87.45**	3.13			2.93		
	NCI-H226	0.94	**90.04**	**73.48**	3.15			2.98		
	NCI-H23	1.79	**98.40**	**85.92**	1.33			1.06		
	NCI-H322M	0.78	**69.16**	59.18	1.78			1.40		
	NCI-H460	−4.28	**92.40**	**90.72**	1.25			0.517		
	NCI-H522	24.81	**139.28**	**128.71**	0.304			0.196		
***Colon Cancer***	COLO 205	−2.26	20.75	17.63	1.96	1.26	0.98	1.98	1.36	0.84
	HCC-2998	−2.95	65.24	61.52	2.27			2.89		
	HCT-116	-	**96.32**	**96.52**	0.386			0.416		
	HCT-15	−3.77	**90.36**	**90.48**	0.417			0.342		
	HT29	19.60	**133.23**	**97.07**	2.66			2.75		
	KM12	0.07	**91.04**	**90.07**	0.501			0.506		
	SW-620	−5.14	**92.65**	**83.27**	0.608			0.659		
***CNS Cancer***	SF-268	1.27	**88.05**	**79.78**	0.651	1.09	1.14	0.621	1.23	0.93
	SF-295	1.78	**84.50**	**76.89**	2.78			3.07		
	SF-539	3.08	**110.32**	**91.36**	0.354			0.38		
	SNB-19	−1.82	**85.25**	**71.02**	1.36			1.41		
	SNB-75	−0.46	**77.26**	**94.88**	0.822			0.994		
	U251	1.88	**91.19**	**83.69**	0.564			0.916		
***Melanoma***	LOX IMVI	1.23	**96.77**	**96.21**	0.55	0.76	1.63	0.497	0.72	1.59
	MALME-3M	−0.37	**92.79**	**87.91**	0.585			0.488		
	M14	−4.24	**93.25**	**95.66**	0.343			0.349		
	MDA-MB-435	−4.44	**91.16**	**112.15**	0.21			0.243		
	SK-MEL-2	12.95	**118.73**	**104.36**	0.661			0.302		
	SK-MEL-28	−8.09	**81.67**	**74.42**	1.12			1.19		
	SK-MEL-5	−7.15	**128.80**	**112.95**	0.834			0.661		
	UACC-257	3.56	**69.55**	58.09	2.21			2.19		
	UACC-62	−2.07	**95.12**	**94.13**	0.326			0.552		
***Ovarian Cancer***	IGROV1	−9.32	**91.57**	**87.46**	0.919	2.10	0.59	1.99	1.41	0.81
	OVCAR-3	−4.55	**104.46**	**86.01**	0.552			0.511		
	OVCAR-4	−7.52	**74.40**	**68.58**	0.845			1.29		
	OVCAR-5	−6.96	**77.89**	43.27	2.75			2.71		
	OVCAR-8	−0.06	**96.74**	**93.48**	2.52			1.59		
	NCI/ADR-RES	−0.60	**79.56**	**78.50**	5.59			0.538		
	SK-OV-3	7.73	**128.58**	**100.16**	1.53			1.26		
***Renal Cancer***	786-0	−3.54	**98.73**	**86.31**	1.06	1.23	1.01	0.634	1.26	0.91
	A498	4.70	**88.83**	**82.44**	2.68			3.82		
	ACHN	−4.23	**82.04**	**81.30**	0.49			0.476		
	CAKI-1	−	**75.80**	**85.21**	0.657			0.818		
	RXF 393	−2.31	**144.52**	**109.98**	1.00			1.27		
	SN 12C	−0.55	**84.29**	**91.91**	0.799			0.935		
	TK-10	12.38	**87.27**	**79.48**	2.91			1.79		
	UO-31	15.23	**99.52**	**91.28**	0.302			0.362		
***Prostate Cancer***	PC-3	−1.38	**91.51**	**89.77**	1.60	1.48	0.84	1.80	1.31	0.87
	DU-145	−5.84	**90.91**	**83.46**	1.37			0.833		
*Breast Cancer*	MCF7	-	**98.70**	**95.79**	0.343	1.29	0.96	0.336	1.28	0.89
	MDA-MB-231	−9.02	**88.67**	**89.85**	2.38			2.38		
	HS 578T	1.56	**77.50**	**89.78**	1.12			1.20		
	BT-549	−4.15	**103.68**	**98.59**	0.571			0.526		
	T-47D	11.07	**87.57**	**85.02**	1.62			1.25		
	MDA-MB-468	−6.31	**101.76**	**87.50**	1.71			1.99		
Mean		0.78	**92.41**	**87.28**						
Total cell line and sum of concentration	60				74.456			68.952		
MID ^a^					1.241			1.149		

(-) Indicates activity not tested; ^a^ The average sensitivity of all cell lines toward the test agent in µM; ^b^ The average sensitivity of all cell lines of a particular subpanel toward the test agent in µM; Bold font shows the good percent growth inhibition (%GI) (≥68%) in one dose assay; %GI = 100 − GP; MID ^a^ and MID ^b^ are the mean GI_50_ of 60 NCI cancer cell lines and the individual cancer cell line panels; Selectivity ratio (SR) = MID ^a^/MID ^b^; GI_50_, stands for 50% growth inhibition.

**Table 4:** The molecular docking results of curcumin analogues against EGFR.

S. No.	Compound	2D Molecular Dockin	Docking Score	Glide Emodel	Interaction
1	**3a**	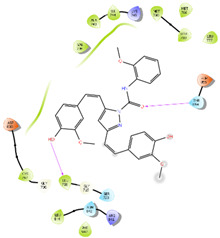	−7.957	−80.016	H-Bond (Leu718); H-Bond (Thr854)
2	**3b**	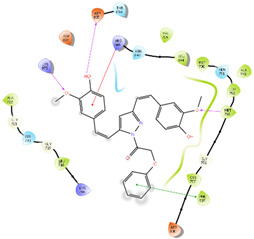	−7.778	−87.340	H-Bond (Met793); H-Bond (Asp855); H-Bond (Lys875); π-π-Stacking (Phe997); π-Cationic (Arg841)
3	**3c**	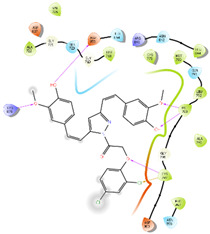	−6.003	−75.919	H-Bond (Met793); H-Bond (Asp855); H-Bond (Lys875); H-Bond (Cys797); Halogen bond (Cys797)
4	**Curcumin**	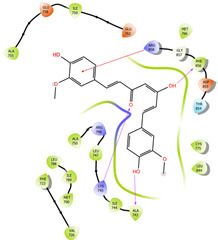	−7.391	−85.784	H-Bond (Phe856); H-Bond (Ala743); H-Bond (Lys745); π-Cationic (Arg858)

## Data Availability

The authors confirm that the data supporting the study’s findings are included in the article and its supplementary materials.

## Supplementary Materials

The following supporting information can be downloaded at: https://www.mdpi.com/article/10.3390/plants11212835/s1, Characterization of compounds **3a–c**; Figure S1: The DFT-optimized conformations of the compounds **3a–c**; Figure S2: Visualization of HOMO/LUMO of compounds **3a–c**; Figures S3 and S4: Antiproliferative profile of compound **3a–c** and curcumin (Cu), Figures S5–S7: Anticancer data of compounds **3a–c** in single dose assay; Figures S8 and S9 Anticancer data of compounds **3b,c** in the five dose assay; Table S1: 60 NCI cancer cell lines based antiproliferative activity of curcumin analogues **3a–c** in single dose (10 µM) and 5-dose assay (0.001–100 µM) of curcumin analogues **3b–c**.

## Abbreviations

CNS	Central nervous system
DFT	Density functional theory
EGFR	Epidermal growth factor receptor
ES-MS	Electrospray Ionization Mass Spectrometry
eV	Electron volt
GI	Growth inhibition
GP	Growth percent
HOMO	highest occupied molecular orbital
IR	Infrared
LC	Lethal concentration
LUMO	lowest unoccupied molecular
NCI	National Cancer Institute
NMR	Nuclear magnetic resonance
NP	Natural product
TGI	Total growth inhibition
TLC	Thin layer chromatography
