# Supplementary material for: Synthesis, DFT Analyses, Antiproliferative Activity, and Molecular Docking Studies of Curcumin Analogues
Source: Plants (Basel). 2022 Oct 25;11(21):2835. doi: 10.3390/plants11212835 (PMC9655326; doi:10.3390/plants11212835)
Supplement: Supplementary file 1 [file plants-11-02835-s001.zip › plants-1932114-supplementary.pdf]

SUPPLEMENTARY INFORMATION

Characterization of compounds (3a-c)

*3,5-Bis(4-hydroxy-3-methylstyryl)-N-(2-methoxylphenyl)-1H-pyrazole-1-carboxamide (**3a**)* Yield 82%; Brown solid (EtOH), mp 150-152 °C; yield 78%; IR (KBr) v_{max}: 3427 (OH), 3299 (NH), 1650 (C=O), 1544 (C=N), 1355 (O-CH_3) cm^{-1}. ^1H NMR (400 MHz, DMSO-d_6): δ ppm 3.69 (3H, s, OCH_3), 3.83 (6H, s, OCH_3), 4.62 (2H, s, CH_2), 6.61 (1H, s, CH=C), 6.73 (2H, d, *J* = 12.4 Hz, CH and CH), 6.75 (2H, d, *J* = 12.4 Hz, CH and CH), 6.80-7.13 (10H, m, ArH), 9.14 (1H, s, ArOH), 9.56 (1H, s, ArOH), 10.56 (1H, s, CONH); ^{13}C NMR (100 MHz, DMSO-d_6): δ ppm 162.91, 156.81, 151.32, 147.21, 144.93, 133.22, 133.41, 128.81, 128.03, 123.60, 120.09, 122.68, 116.82, 114.07, 107.73, 56.24; EIMS *m/z* = 514.1 $[M+1]^+$.

*3,5-Bis(4-hydroxy-3-methoxystyryl)-1H-pyrazole-1-yl-(phenoxy)ethanone (**3b**)* Yield, 80%; Light brown solid, mp 140-142 °C; IR (KBr) v_{max}: 3431 (OH), 1600 (C=O), 1548 (C=N), 1514 (C=C), 1280 (C-O) cm^{-1}. ^1H NMR (400 MHz, DMSO-d_6): δ ppm 3.83 (6H, s, OCH_3), 4.60 (2H, s, CH_2), 6.61 (1H, s, CH=C), 6.75 (2H, d, *J* = 14.2 Hz, CH and CH), 6.77 (2H, d, *J* = 14.4 Hz, CH and CH), 6.83-7.56 (11H, m, ArH), 9.14 (1H, s, ArOH), 9.64 (1H, s, ArOH); ^{13}C NMR (100 MHz, DMSO-d_6): δ ppm 160.71, 151.32, 147.46, 144.89, 137.31, 133.41, 129.83, 128.81, 123.6, 121.18, 120.19, 116.80, 114.31, 112.101, 107.72, 72.09, 56.41; EIMS *m/z* = 499.51 $[M+1]^+$.

*3,5-Bis(4-hydroxy-3-methoxystyryl)-1H-pyrazole-1-yl-(2,4-dichlorophenoxy)ethanone (**3c**)* Yield 78%; Light yellow solid, mp 102-104 °C; yield 74%; IR (KBr) v_{max}: 3427 (OH), 1610 (C=O), 1585 (C=N), 1548 (C=C), 1276 (C-O), 680 (C-Cl) cm^{-1}. ^1H NMR (400 MHz, DMSO-d_6): δ ppm 3.82 (6H, s, OCH_3), 4.80 (2H, s, CH_2), 6.61 (1H, s, CH=C), 6.75 (2H, d, *J* = 14.4 Hz, CH and CH), 6.77 (2H, d, *J* = 14.4 Hz, CH and CH), 6.91-7.59 (9H, m, ArH), 9.15 (1H, s, ArOH), 9.91 (1H, s, ArOH); ^{13}C NMR (100 MHz, DMSO-d_6): δ ppm 162.71, 152.81, 151.32, 147.21, 144.89, 133.41, 133.01, 128.81, 128.03, 124.60, 120.18, 117.13, 116.81, 112.01, 107.71, 72.24, 56.25; EIMS *m/z* = 567.41 $[M]^+$, 569.4 $[M+2]^+$.

3a

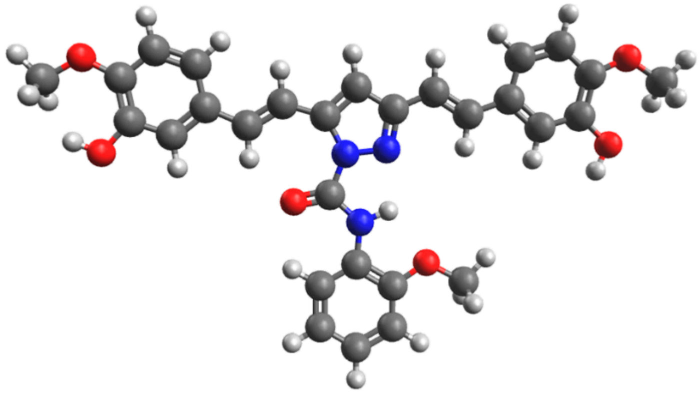

3b

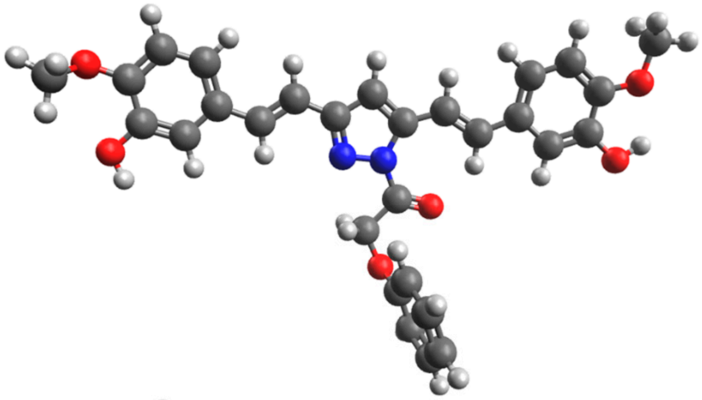

3c

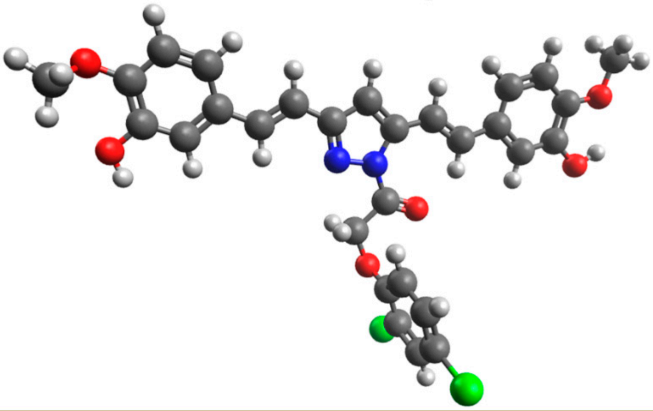

Figure S1. The DFT-optimized conformations of the compounds **3a–c**.

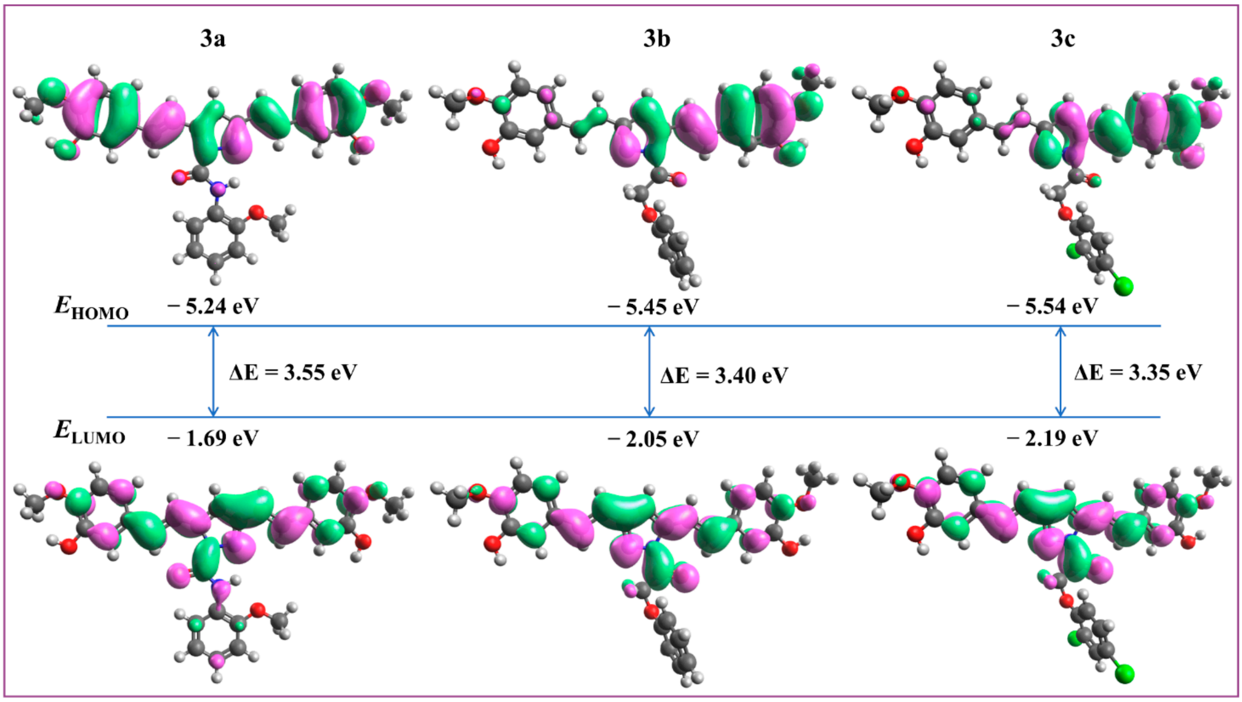

Figure S2. Visualization of HOMO/LUMO of compounds **3a–c**.

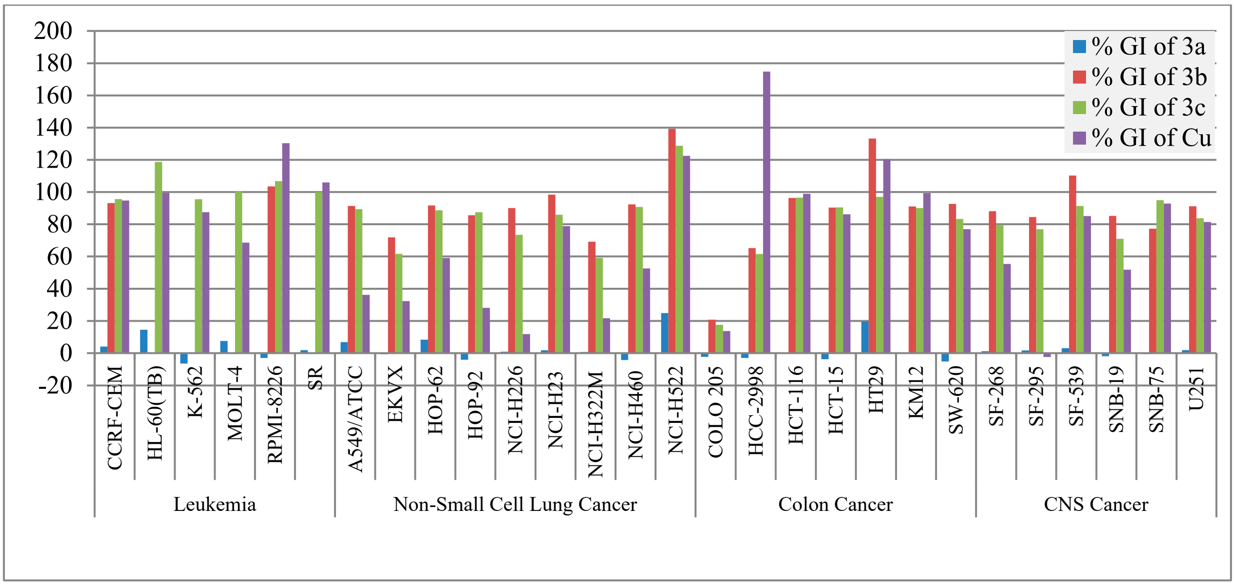

Figure S3. The comparative %GIs of curcumin analogues (**3a-c**) and curcumin (Cu) against leukemia, non-small lung cancer, colon and CNS cancer panels at 10 μM.

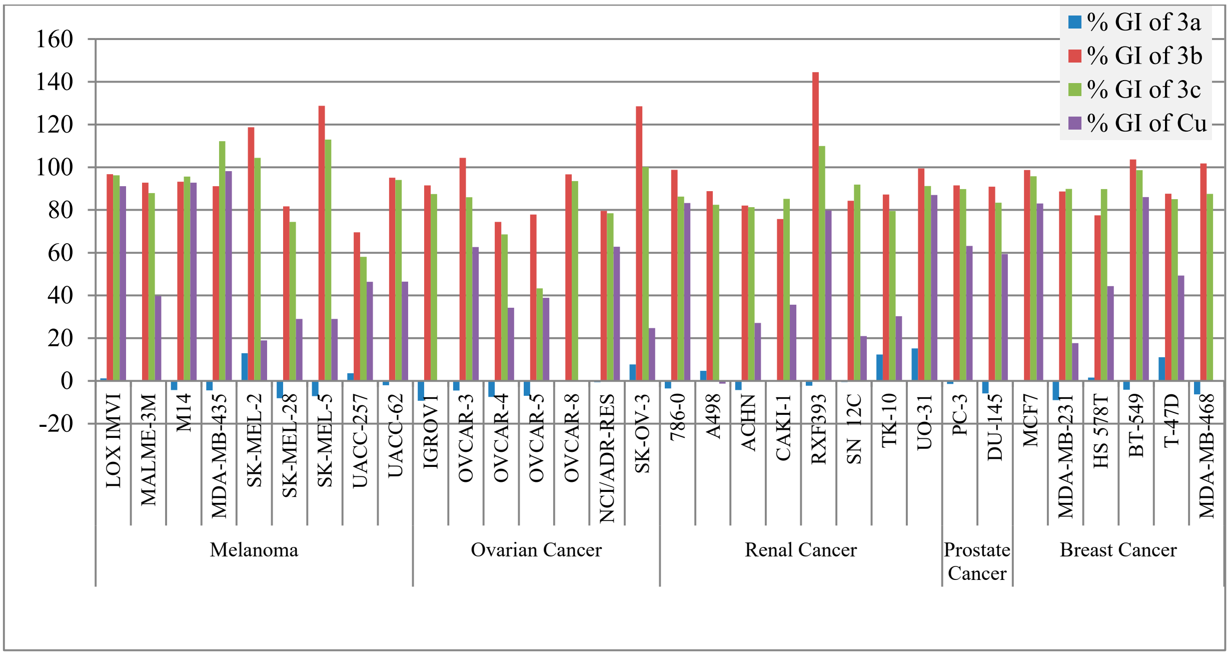

Figure S4. The comparative %GIs of curcumin analogues (**3a-c**) and curcumin (Cu) against melanoma, ovarian, renal, prostate and breast cancer panels at 10 µM.

Table S1: 59 NCI cancer cell lines based antiproliferative activity of curcumin analogues **3a-c** in single dose (10 µM) and 5-dose assay (0.001-100 µM) of curcumin analogues **3b-c**.

**Panel**	**Cell line**	**One dose assay (10 µM)**			**Five dose assay**			
		**3a**	**3b**	**3c**	**3b**		**3c**	
		GP	GP	GP	TGI	LC_{50}	TGI	LC_{50}
Leukemia	CCRF-CEM	95.87	6.79	4.35	1.34	>100	>100	>100
	HL-60(TB)	85.39	-	-18.70	0.901	>100	1.31	>100
	K-562	106.41	-	4.41	1.08	>100	>100	>100
	MOLT-4	92.36	-	-0.33	3.26	>100	3.46	>100
	RPMI-8226	102.92	-3.54	-6.80	2.36	>100	7.62	>100
	SR	98.12	-	0.01	2.46	>100	-	>100
Non-Small Cell Lung Cancer	A549/ATCC	93.11	8.68	10.58	11.1	47.2	10.3	54.2
	EKVX	100.71	28.12	38.35	10.3	32.4	9.72	36.2
	HOP-62	91.63	8.39	11.28	4.95	33.3	3.16	26.7
	HOP-92	104.08	14.37	12.55	20.4	>100	7.94	36.5
	NCI-H226	99.06	9.96	26.52	9.94	>100	10.2	>100
	NCI-H23	98.21	1.60	14.08	6.64	>100	5.00	>100
	NCI-H322M	99.22	30.84	40.82	10.3	46.2	11.3	56.0
	NCI-H460	104.28	7.60	9.28	10.2	49.2	14.2	>100
	NCI-H522	73.19	-39.28	-28.71	0.97	10.8	0.837	24.9

Colon Cancer	COLO 205	102.26	79.25	82.37	3.95	7.94	4.01	8.12
	HCC-2998	102.95	34.76	38.48	10.2	32.0	12.4	37.5
	HCT-116	-	3.68	3.48	10	>100	6.21	53.1
	HCT-15	103.77	9.64	9.52	7.96	35.1	3.61	53.8
	HT29	80.40	-33.23	2.93	6.35	44.7	7.03	>100
	KM12	99.93	8.96	9.93	6.81	51.7	26.2	>100
	SW-620	105.14	7.35	16.73	9.02	82.3	>100	>100
CNS Cancer	SF-268	98.73	11.95	20.22	5.65	29.4	9.84	>100
	SF-295	98.22	15.50	23.11	7.70	38.5	8.87	41.6
	SF-539	96.92	-10.32	8.64	2.47	32.5	2.85	25.4
	SNB-19	101.82	14.75	28.98	9.59	61.7	11.7	43.5
	SNB-75	100.46	22.74	5.12	5.30	40.2	7.81	>100
	U251	98.12	8.81	16.31	5.96	28.0	6.15	30.8
Melanoma	LOX IMVI	98.77	3.23	3.79	3.29	>100	3.29	57.1
	MALME-3M	100.37	7.21	12.09	3.71	33.8	5.03	35.1
	M14	104.24	6.75	4.34	2.68	28.6	4.83	35.9
	MDA-MB-435	104.44	8.84	-12.15	0.492	46.9	14.7	>100
	SK-MEL-2	87.05	-18.73	-4.36	3.56	23.0	1.54	34.9
	SK-MEL-28	108.09	18.37	26.56	10.4	>100	7.32	41.4
	SK-MEL-5	107.15	-28.80	-12.95	3.52	16.8	3.03	15.3
	UACC-257	96.54	30.45	41.91	7.34	27.3	7.13	26.8
	UACC-62	102.07	4.88	5.87	1.27	8.65	12.0	>100
Ovarian Cancer	IGROV1	109.32	8.43	12.54	8.00	>100	10.3	>100
	OVCAR-3	104.55	-4.46	13.99	2.77	>100	-	>100
	OVCAR-4	107.52	25.60	31.42	12.3	>100	>100	>100
	OVCAR-5	106.96	22.11	56.73	6.81	>100	8.32	>100
	OVCAR-8	100.06	3.26	6.52	8.57	35.2	6.09	>100
	NCI/ADR-RES	100.60	20.44	21.50	11.7	>100	21.1	>100
	SK-OV-3	92.27	-28.58	-0.16	3.45	7.75	3.43	9.35
Renal Cancer	786-0	103.54	1.27	13.69	3.58	13.6	2.52	8.35
	A498	95.30	11.17	17.56	6.49	21.8	12.0	35.8
	ACHN	104.23	17.96	18.70	11.1	>100	9.13	44.1
	CAKI-1	-	24.20	14.79	12.4	>100	>100	>100
	RXF 393	102.31	-44.52	-9.98	2.52	6.34	3.33	8.77
	SN 12C	100.45	15.71	8.09	7.66	>100	65.8	>100
	TK-10	87.62	12.73	20.52	10.9	33.0	8.06	29.2
	UO-31	84.77	0.48	8.72	4.12	26.7	2.30	11.4
Prostate	PC-3	101.38	8.49	10.23	>100	>100	8.02	>100

Cancer	DU-145	105.84	9.09	16.54	8.06	29.4	9.94	32.2
Breast Cancer	MCF7	-	1.30	4.21	2.83	4.48	2.33	94.8
	MDA-MB-231	109.02	11.33	10.15	15.3	>100	23.4	>100
	HS 578T	98.44	22.50	10.22	6.84	>100	>100	>100
	BT-549	104.15	-3.68	1.41	4.03	>100	3.34	19.2
	T-47D	88.93	12.43	14.98	6.95	50.9	3.99	>100
	MDA-MB-468	106.31	-1.76	12.50	7.39	38.5	5.34	25.3
Mean		99.22	7.59	12.72				

(-) Indicates activity not tested; [a] The average sensitivity of all cell lines toward the test agent in μM; [b] The average sensitivity of all cell lines of a particular subpanel toward the test agent in μM; Bold font showed the good growth percent (GP) (≤32%) in one dose assay; LC_{50} and TGI stand for 50% lethal concentration, and total growth inhibition respectively.

Developmental Therapeutics Program One Dose Mean Graph	NSC: D-793120 / 1	Conc: 1.00E-5 Molar	Test Date: Sep 12, 2016
	Experiment ID: 1609OS82		Report Date: Sep 29, 2016

Panel/Cell Line	Growth Percent
Leukemia	
CCRF-CEM	95.87
HL-60(TB)	85.39
K-562	106.41
MOLT-4	92.36
RPMI-8226	102.92
SR	98.12
Non-Small Cell Lung Cancer	
A549/ATCC	93.11
EKVX	100.71
HOP-62	91.63
HOP-92	104.08
NCI-H226	99.06
NCI-H23	98.21
NCI-H322M	99.22
NCI-H460	104.28
NCI-H522	73.19
Colon Cancer	
COLO 205	102.26
HCC-2998	102.95
HCT-15	103.77
HT29	80.40
KM12	99.93
SW-620	105.14
CNS Cancer	
SF-268	98.73
SF-295	98.22
SF-539	96.92
SNB-19	101.82
SNB-75	100.46
U251	98.12
Melanoma	
LOX IMVI	98.77
MALME-3M	100.37
M14	104.24
MDA-MB-435	104.44
SK-MEL-2	87.05
SK-MEL-28	108.09
SK-MEL-5	107.15
UACC-257	96.54
UACC-62	102.07
Ovarian Cancer	
IGROV1	109.32
OVCAR-3	104.55
OVCAR-4	107.52
OVCAR-5	106.96
OVCAR-8	100.06
NCI/ADR-RES	100.60
SK-OV-3	92.27
Renal Cancer	
786-0	103.54
A498	95.30
ACHN	104.23
RXF 393	102.31
SN12C	100.45
TK-10	87.62
UO-31	84.77
Prostate Cancer	
PC-3	101.38
DU-145	105.84
Breast Cancer	
MDA-MB-231/ATCC	109.02
HS 578T	98.44
BT-549	104.15
T-47D	88.93
MDA-MB-468	106.31
Mean	99.22
Delta	26.03
Range	36.13

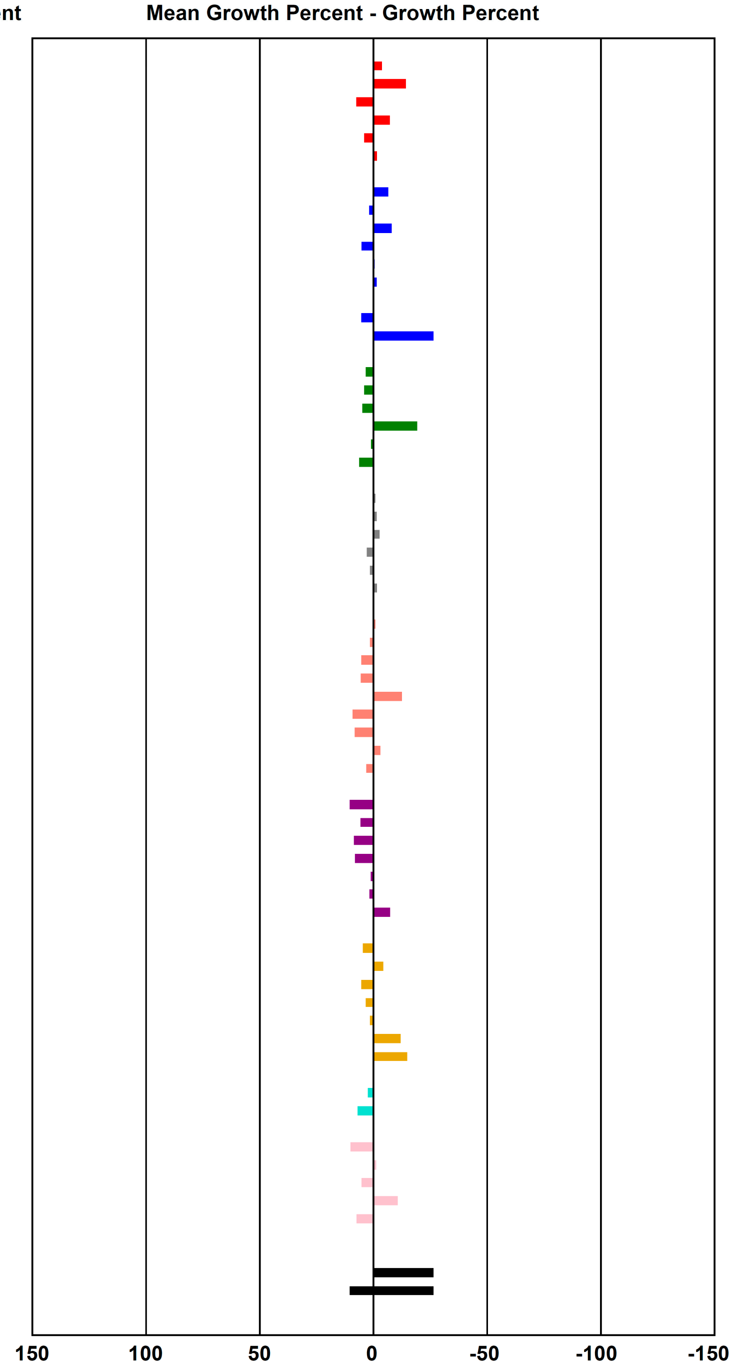

Figure S5. The anticancer data of compound **3a** at 10 μM.

**Developmental Therapeutics Program**	**NSC:** D-782199 / 1	**Conc:** 1.00E-5 Molar	**Test Date:** Oct 06, 2014
**One Dose Mean Graph**	**Experiment ID:** 1410OS65		**Report Date:** Oct 23, 2014

**Panel/Cell Line**	**Growth Percent**
Leukemia	
CCRF-CEM	6.79
RPMI-8226	-3.54
Non-Small Cell Lung Cancer	
A549/ATCC	8.68
EKVX	28.12
HOP-62	8.39
HOP-92	14.37
NCI-H226	9.96
NCI-H23	1.60
NCI-H322M	30.84
NCI-H460	7.60
NCI-H522	-39.28
Colon Cancer	
COLO 205	79.25
HCC-2998	34.76
HCT-116	3.68
HCT-15	9.64
HT29	-33.23
KM12	8.96
SW-620	7.35
CNS Cancer	
SF-268	11.95
SF-295	15.50
SF-539	-10.32
SNB-19	14.75
SNB-75	22.74
U251	8.81
Melanoma	
LOX IMVI	3.23
MALME-3M	7.21
M14	6.75
MDA-MB-435	8.84
SK-MEL-2	-18.73
SK-MEL-28	18.37
SK-MEL-5	-28.80
UACC-257	30.45
UACC-62	4.88
Ovarian Cancer	
IGROV1	8.43
OVCAR-3	-4.46
OVCAR-4	25.60
OVCAR-5	22.11
OVCAR-8	3.26
NCI/ADR-RES	20.44
SK-OV-3	-28.58
Renal Cancer	
786-0	1.27
A498	11.17
ACHN	17.96
CAKI-1	24.20
RXF 393	-44.52
SN12C	15.71
TK-10	12.73
UO-31	0.48
Prostate Cancer	
PC-3	8.49
DU-145	9.09
Breast Cancer	
MCF7	1.30
MDA-MB-231/ATCC	11.33
HS 578T	22.50
BT-549	-3.68
T-47D	12.43
MDA-MB-468	-1.76
Mean	7.59
Delta	52.11
Range	123.77

Mean Growth Percent - Growth Percent

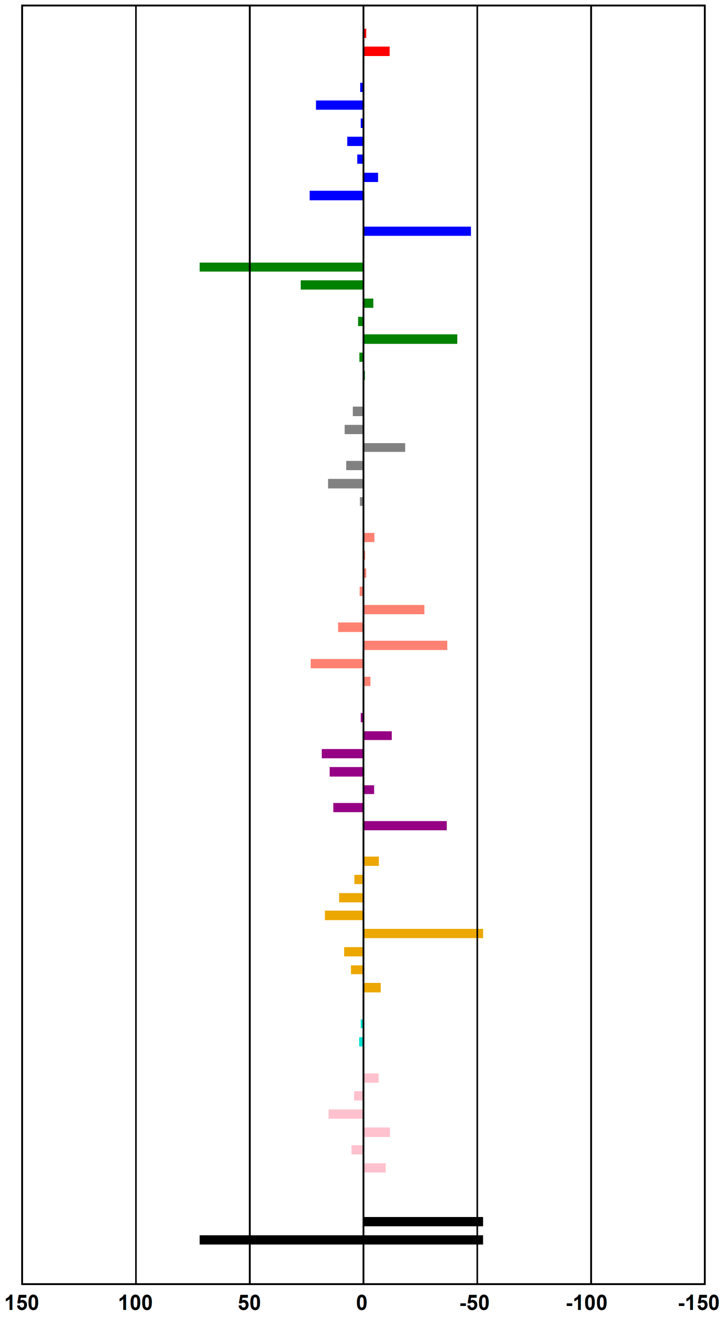

Figure S6. The anticancer data of compound **3b** at 10 μM.

Developmental Therapeutics Program One Dose Mean Graph	NSC: D-782200 / 1	Conc: 1.00E-5 Molar	Test Date: Oct 06, 2014
	Experiment ID: 1410OS65		Report Date: Oct 23, 2014

Panel/Cell Line	Growth Percent
**Leukemia**	
CCRF-CEM	4.35
HL-60(TB)	-18.70
K-562	4.41
MOLT-4	-0.33
RPMI-8226	-6.80
SR	0.01
**Non-Small Cell Lung Cancer**	
A549/ATCC	10.58
EKVX	38.35
HOP-62	11.28
HOP-92	12.55
NCI-H226	26.52
NCI-H23	14.08
NCI-H322M	40.82
NCI-H460	9.28
NCI-H522	-28.71
**Colon Cancer**	
COLO 205	82.37
HCC-2998	38.48
HCT-116	3.48
HCT-15	9.52
HT29	2.93
KM12	9.93
SW-620	16.73
**CNS Cancer**	
SF-268	20.22
SF-295	23.11
SF-539	8.64
SNB-19	28.98
SNB-75	5.12
U251	16.31
**Melanoma**	
LOX IMVI	3.79
MALME-3M	12.09
M14	4.34
MDA-MB-435	-12.15
SK-MEL-2	-4.36
SK-MEL-28	26.56
SK-MEL-5	-12.95
UACC-257	41.91
UACC-62	5.87
**Ovarian Cancer**	
IGROV1	12.54
OVCAR-3	13.99
OVCAR-4	31.42
OVCAR-5	56.73
OVCAR-8	6.52
NCI/ADR-RES	21.50
SK-OV-3	-0.16
**Renal Cancer**	
786-0	13.69
A498	17.56
ACHN	18.70
CAKI-1	14.79
RXF 393	-9.98
SN12C	8.09
TK-10	20.52
UO-31	8.72
**Prostate Cancer**	
PC-3	10.23
DU-145	16.54
**Breast Cancer**	
MCF7	4.21
MDA-MB-231/ATCC	10.15
HS 578T	10.22
BT-549	1.41
T-47D	14.98
MDA-MB-468	12.50
Mean	12.72
Delta	41.43
Range	111.08

Mean Growth Percent - Growth Percent

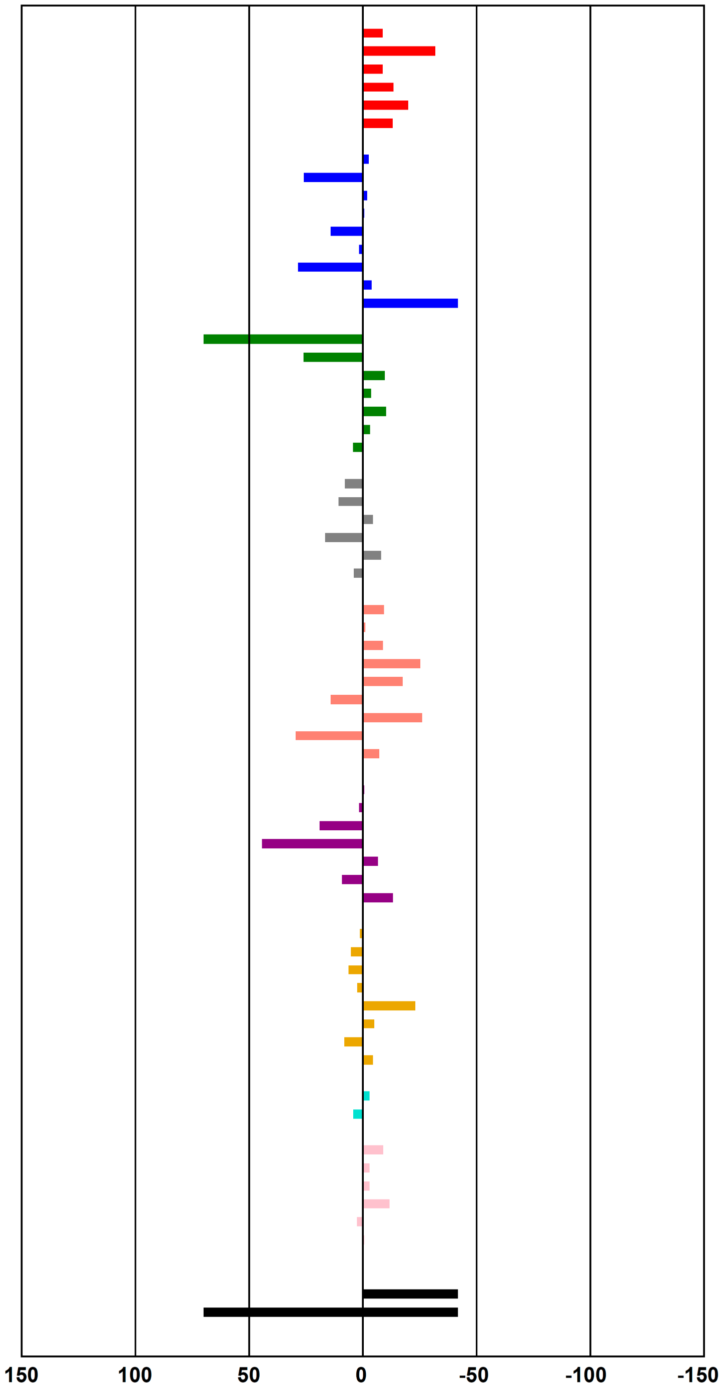

Figure S7. The anticancer data of compound **3c** at 10 μM.

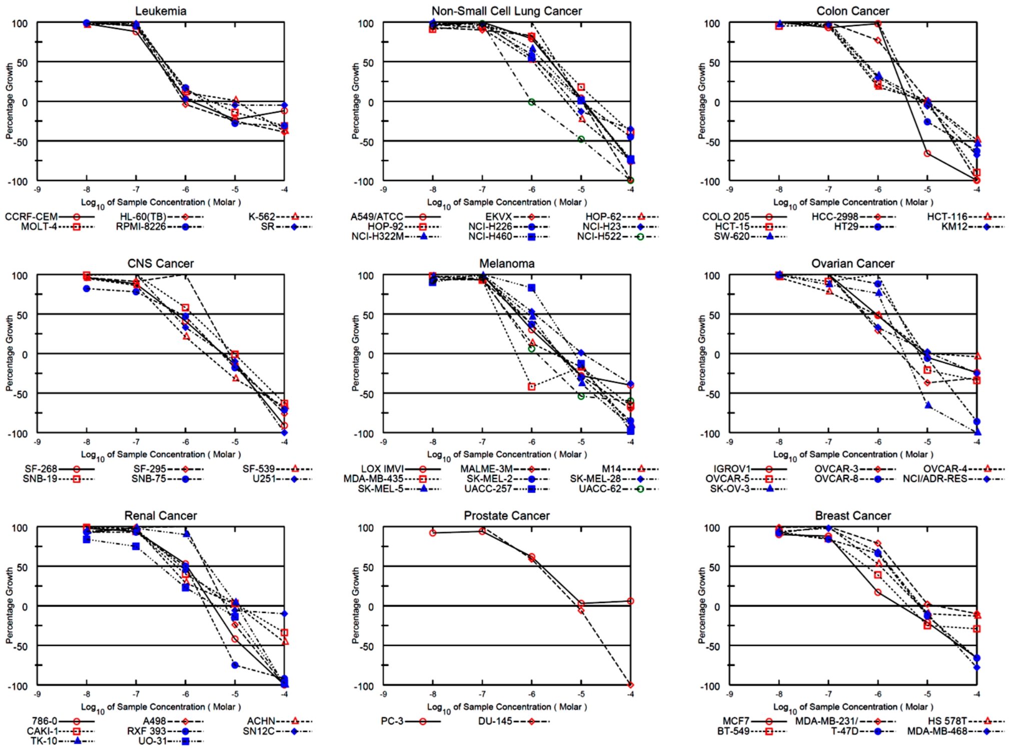

Figure S8. 60 NCI cancer cell lines based antiproliferative activity of curcumin analogue **3b.**

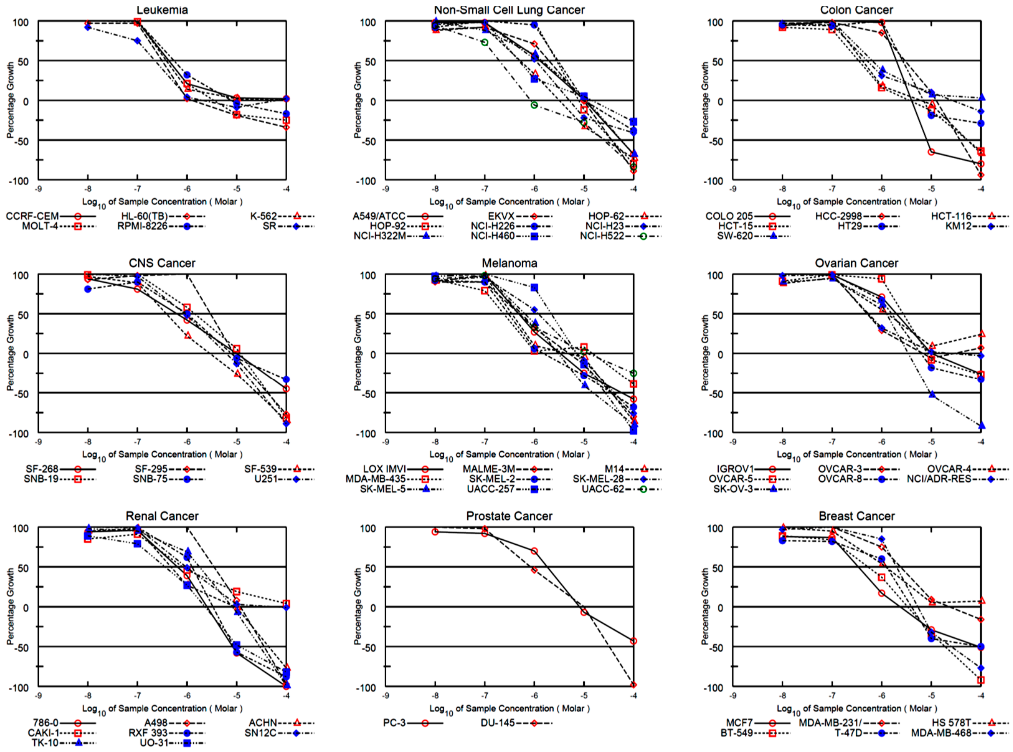

Figure S9. 60 NCI cancer cell lines based antiproliferative activity of curcumin analogue **3c.**
